# Regional Differences in Young’s Modulus of the Porcine Lens Capsule

**DOI:** 10.1007/s10439-026-04012-0

**Published:** 2026-02-14

**Authors:** Hoyeon Jang, Myles Cline, Jeongjin Lee, Matthew Reilly, Hanna Cho

**Affiliations:** 1 Department of Biomedical Engineering, The Ohio State University, Columbus, Ohio 43210 USA; 2 Division of Biostatistics, The Ohio State University, Columbus, Ohio 43210 USA; 3 Department of Mechanical and Aerospace Engineering, The Ohio State University, Columbus, Ohio 43210 USA

**Keywords:** Atomic force microscopy (AFM), Lens capsule, Young’s modulus, Porcine eye, Biomechanics/Ocular biomechanics

## Abstract

**Purpose:**

The ocular lens capsule is a biomechanically specialized basement membrane essential for lens function, yet its regional micromechanical properties remain incompletely characterized.

**Methods:**

We employed atomic force microscopy (AFM)-based force spectroscopy to map the stiffness of young porcine anterior and posterior lens capsule samples under physiologically hydrated conditions. A refined dissection protocol was used to preserve native curvature and hydration, with the anterior and posterior regions isolated via selective capsular puncture. Force–indentation measurements were performed using calibrated silicon cantilevers and analyzed with the Johnson–Kendall–Roberts (JKR) model to extract local Young’s modulus.

**Results:**

Results from over 12,000 force curves revealed that the anterior capsule exhibited significantly higher stiffness (mean 67.9 kPa, standard deviation 40.1 kPa) than the posterior (mean 54.1 kPa, standard deviation 25.2 kPa; *p* < 0.0001), with a wider range of stiffness values. AFM topography confirmed comparable surface morphology, ruling out roughness as a confounding factor.

**Conclusion:**

These findings highlight the functional specialization of the lens capsule and the utility of AFM for high-resolution biomechanical characterization. These measurement techniques will be applied to human lens capsules to elucidate age-related changes in capsule properties pertaining to presbyopia, inform surgical strategies, lens capsule modeling, and the design of accommodative intraocular lenses.

**Supplementary Information:**

The online version contains supplementary material available at 10.1007/s10439-026-04012-0.

## Introduction

The lens capsule is a thin, elastic basement membrane that envelops the crystalline lens and plays a crucial role in ocular physiology. It facilitates accommodation, preserves lens transparency, and mediates metabolic exchange with surrounding ocular fluids acting as a selective, semipermeable barrier [1, 2]. Structurally, the capsule is a dense extracellular matrix primarily composed of type IV collagen, laminin, nidogen, and perlecan, arranged to enable large elastic deformation while preserving optical clarity [3, 4].

Beyond its physiological roles, the biomechanical integrity of the lens capsule is central to the success of ophthalmic procedures such as cataract surgery. In particular, the anterior capsule must withstand substantial mechanical manipulation during continuous curvilinear capsulorhexis (CCC) and intraocular lens (IOL) implantation. Inadequate mechanical performance can lead to complications such as radial tears or capsular contraction syndrome [5, 6]. Despite its clinical importance, few studies have investigated the micromechanical properties of the capsule, particularly under physiologically hydrated conditions [7].

The stiffness of the lens capsule is also fundamental to understanding the biomechanics of accommodation and the pathophysiology of presbyopia which is the loss of near-sightedness with age. During accommodation, the capsule transmits ciliary muscle forces to the lens substance, enabling dynamic shape change that modulates optical power [8–10]. Aging leads to a progressive stiffening of the capsule and lens, reducing their ability to deform and thereby diminishing accommodative amplitude which is a hallmark of presbyopia [1, 10]. Regional differences in capsule stiffness may significantly influence how these forces are distributed, impacting the overall shape transformation of the lens [11]. Precise characterization of these regional properties is therefore essential for refining computational models of accommodation, which depend critically on accurate mechanical parameters [12, 13]. Additionally, experimental studies suggest that interventions targeting the biomechanical properties of the capsule, such as lens refilling or accommodative intraocular lenses, require detailed knowledge of local stiffness variations to be effective [14, 15]. Collectively, these insights emphasize the need for spatially resolved, physiologically relevant stiffness measurements [16, 17] to advance both mechanistic understanding and therapeutic strategies in ophthalmology.

Anatomical and structural distinctions suggest that the anterior capsule should be stiffer than the posterior. The anterior capsule thickens with age due to matrix production by underlying epithelial cells, contributing to increased stiffness and structural complexity [1, 2]. Conversely, the posterior capsule, which lacks epithelial coverage, remains thin and relatively compliant after early development [3, 18]. While existing studies support such anterior–posterior difference in stiffness [19, 20], few have captured this contrast with high spatial resolution while maintaining physiological hydration [21]. Identifying these regional mechanical differences at micro-scales is not only scientifically intriguing but may also inform surgical strategy and biomaterial design [22].

Traditional measurement techniques, including tensile testing, inflation testing, indentation methods, and atomic force microscopy (AFM), have been employed to characterize the biomechanical behavior of the capsule [5, 19, 23, 24]. However, these approaches often involve tissue dehydration, flattening, or large-scale averaging, which likely alter matrix mechanics compared to the native, curved, and hydrated tissue environment [3, 18]. Moreover, reported values for key parameters such as Young’s modulus and ultimate tensile stress differ dramatically across studies, owing to variation in species, anatomical regions, age, and measurement methods. For example, Young’s modulus estimates for the anterior human lens capsule range from ~ 0.3 to over 2 MPa in tensile testing studies [18, 25], while AFM measurements in human and primate tissues have yielded values from ~ 30 kPa to over 800 kPa [19, 26]. Similar discrepancies have been reported in other soft biological tissues, where heterogeneity in composition and structure leads to wide ranges of measured stiffness values, as seen in breast tissue [27] and porcine cartilage [28]. These findings highlight the inherent challenge of characterizing compliant, heterogeneous matrices and further support the need for microscale approaches capable of resolving local mechanical variation under physiological conditions. These various values, summarized in Table 1, highlight the absence of standardization and underscore the importance of measuring mechanical properties at the microscale under hydrated, physiologically relevant conditions. Table 1 has been reorganized and updated from the comprehensive review by Avetisov et al. [20] to compare Young’s modulus of lens capsule across species, methods, and anatomical regions. Similar challenges have been observed in other soft biological systems, where tissue heterogeneity strongly influences measured stiffness values, as demonstrated in breast tissue [27] and porcine cartilage [28], underscoring the importance of using microscale techniques capable of resolving local variation.

**Table 1.** Summary of published studies reporting mechanical properties of the lens capsule across species, anatomical regions, age groups, and experimental methods

Method	Mechanical property	Value [MPa]	Author(s)
Inflation testing/ pressure loading	Young’s modulus	5, Human	Fisher [8], 1969
		0.9, Cat	
		2.5, Rabbit	
		6, Human (Infancy)	Fisher [9], 1969
		2, Human (Old age)	
		0.82 ± 0.29 (at low stress), Cat	Fisher and Wakely [12], 1976
		7.74 ± 1.38 (at rupture), Cat	Fisher and Wakely [12], 1976
		0.56 ± 0.38 (at low stress), Rat	Fisher and Hayes [13], 1979
		11.3 ± 1.9 (at rupture), Rat	Fisher and Hayes [13], 1979
	Ultimate stress	2.3, Human (<20 yrs)	Fisher [9], 1969
		0.7, Human (>70 yrs)	
		17.4 ± 1.6, Cat	Fisher and Wakely [12], 1976
		2.88 ± 0.45, Rat	Fisher and Hayes [13], 1979
Inflation testing	Stiffness	c = 1.07 N/m, Human / Anterior	Pedrigi et al. [5], 2007
Tensile testing	Ultimate stress	17.5 – 1.5, Human / Anterior (7 months-98 yrs)	Krag et al. [41], 1997
		16.1 – 1.1, Human / Posterior	Krag and Andreassen [18], 2003
	Tangent modulus	44.8 – 4.4, Human / Anterior (7 months-98 yrs)	Krag et al. [41], 1997
	Stiffness	c = 1.07 ± 0.11 N/m, Human / Anterior (Normal, near CCC)	Pedrigi et al. [14], 2009
		c = 4.52 ± 1.21 N/m, Human / Anterior (Post-cataract, near CCC)	
		c = 1.07 ± 0.06 N/m, Human / Anterior (Normal, Periphery)	
		c = 1.26 ± 0.32 N/m, Human / Anterior (Post-cataract, Periphery)	
	Young’s modulus	0.3 – 2.3 (0-10% strain), Human / Anterior	Krag and Andreassen [25], 2003
		0.3 – 2.3 (0-10% strain), Human / Posterior	Krag and Andreassen [18], 2003
		0.54 ± 0.03, Rat	Danielsen [23], 2004
		1.2 ± 0.08, Cow	
		1.26 ± 0.06, Sow	
		2.4 ± 0.27, Human	
	Cauchy stress	~ 100 kPa (Finite element analysis), Human / Anterior	Berggren et al.[15], 2021
Optical coherence elastography (OCE)	Young’s modulus	6.33 ± 0.36 kPa, Human (young)	Shi et al. [42], 2024
		13.33 ± 0.74 kPa, Human (mature)	
		1.89 ± 0.8, Porcine / Anterior (6 months)	Feng et al. [43], 2025
		1.32 ± 0.3, Porcine / Posterior (6 months)	
AFM/ Nanoindenter	Young’s modulus	33.7 ± 33.1 kPa, Cynomolgus Monkey (5.9-8 yrs)	Ziebarth et al. [19], 2011
		33.3 ± 16.7 kPa, Baboon (2.8-10.1 yrs)	
		28.7 ± 6.7 kPa, Human (33-41 yrs)	
		47.7 ± 10.4 kPa, Human (53-67 yrs)	
		86.5 ± 37.6 kPa, Human (71-79 yrs)	
		~ 120 kPa Human / Epithelial	Halfter et al. [44], 2013
		~ 40 kPa Human / Vitreal	
		170-830 kPa Human / Anterior	Tsaousis et al.[26], 2014
		34 ± 13 kPa Human / Anterior (45-68 yrs)	Efremov et al.[45], 2020
		61 ± 18 kPa Human / Anterior (45-68 yrs)	
		75 ± 45 kPa Human / Anterior (69-83 yrs)	
		75 ± 18 kPa Human / Anterior (69-83 yrs)	

Values are reported with means and standard deviations or ranges where available. This table is updated and reorganized based on the review by Avetisov et al. (2020) [20]

Atomic force microscopy (AFM)-based force spectroscopy offers a powerful method to address these limitations. Conducted in physiologically relevant liquid environments and supported by contact mechanics models, AFM enables localized stiffness mapping of soft biological tissues with nanometer precision [29, 30]. Its capability to measure minute forces and deformations makes it especially suited for probing compliant, hydrated specimens such as biological membranes. For example, AFM has been applied to quantify micromechanical differences in human lung epithelial cells under pathological and healthy conditions [31], map viscoelastic properties in neural tissues for neurodegenerative disease studies [32], and characterize the stiffness of corneal and retinal tissues to assess age- and disease-related biomechanical changes [19, 33]. AFM has also been successfully applied to other ECM-rich ocular tissues such as Bowman’s membrane of the cornea, where fractal surface analyses revealed microstructural complexity [34]. More broadly, studies of extracellular matrix mechanics emphasize that structure–function relationships at the nanoscale play critical roles in tissue behavior [35], reinforcing the relevance of AFM for investigating the lens capsule as a specialized basement membrane. Moreover, AFM-based approaches have been used to evaluate biomechanical responses in tumor microenvironments, monitor cytoskeletal remodeling in response to external stimuli, and probe adhesion forces in individual protein–ligand interactions [8, 9, 36]. These broad applications across cellular, subcellular, and tissue scales illustrate the versatility of AFM in resolving structural–mechanical relationships in biological systems.

In this study, we use AFM-based force spectroscopy to map the micromechanical properties of anterior and posterior regions of fresh porcine lens capsules under fully hydrated conditions. We aim to determine whether a consistent anterior–posterior stiffness difference exists at the micrometer scale, evaluate intra-regional mechanical heterogeneity, and assess reproducibility across biological replicates. By incorporating a rigorously calibrated AFM workflow and analyzing thousands of localized measurements, this work seeks to uncover intrinsic mechanical differences that reflect functional specialization of the lens capsule. Building on our previous works in applying AFM to diverse biological systems, including probing cell–substrate interactions, nanomechanical bone characterization, collagen matrix electromechanics, and virus-induced structural changes [37–40], we extend this expertise to the ocular lens capsule whose regional mechanical properties have been comparatively underexplored despite their critical roles. These findings have important implications for understanding lens biomechanics, refining surgical strategies, and guiding the design of advanced intraocular lenses and ophthalmic biomaterials.

## Materials and Methods

### Sample Preparation

Porcine eyes (*n* = 13) were obtained from a local abattoir and dissected within 2 hours of post-mortem to preserve tissue viability and hydration status. For anterior capsule preparation, the cornea and iris were carefully excised, and the globe was bisected equatorially to expose the lens. Following vitreous removal, the posterior lens capsule was gently punctured with a fine needle to allow removal of the lens cortex and nucleus without damaging the anterior capsule. The resulting structure consisted of a scleral ring with the anterior lens capsule intact and supported by the zonular fibers. For posterior capsule samples, the anterior capsule was pierced to extract the cortex and nucleus, leaving the posterior capsule attached to the sclera via the zonules. This symmetric dissection ensured that both anterior and posterior samples were collected using the same protocol, differing only in the site of initial capsular perforation. As eyes were acquired in pairs, each anterior and posterior samples were obtained from same porcine. After dissection, 8 mm circular samples were isolated using a sterile biopsy punch and mounted capsule-side up on 15 mm metallic AFM disks (Ted Pella Inc.) via surface tension. All samples remained submerged in chilled phosphate-buffered saline (PBS) and were measured the same day to maintain hydration and minimize degradation.

### AFM Measurement

**Fig. 1:**
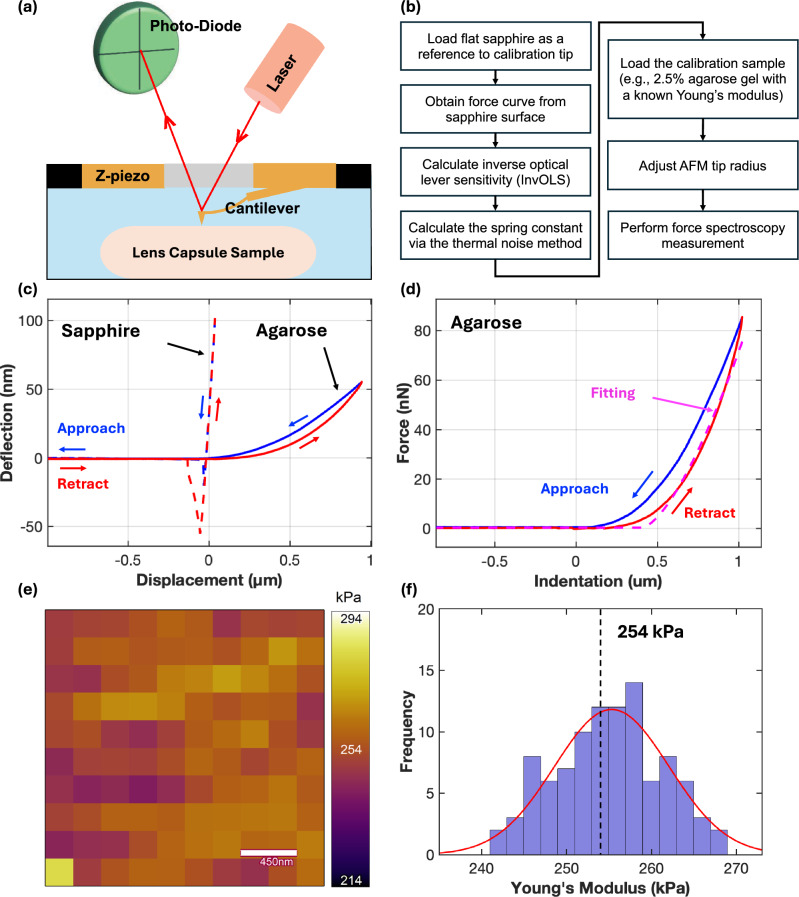
AFM setup and calibration workflow for mechanical characterization of the lens capsule. **a** Schematic of AFM-based force spectroscopy in liquid environment, showing PBS immersion and laser-photodiode deflection detection system. **b** Two-step calibration workflow using InvOLS determination on sapphire and tip radius calibration on 2.5% agarose gel with known Young’s modulus (~254 kPa). **c** Representative deflection–displacement curves for stiff sapphire (steep slope) and soft agarose (gentler response). **d** Force–indentation curve for agarose with retraction segment fitted using the Johnson–Kendall–Roberts (JKR) contact mechanics model. **e** 10 × 10 grid-based force map (2 µm × 2 µm area) on agarose sample demonstrating spatial calibration consistency. **f** Histogram of extracted Young’s modulus values from the force map showing Gaussian-like distribution centered at ~254 kPa

AFM-based force spectroscopy enables quantitative measurements of microscale mechanical properties in soft, hydrated biological samples by recording the force–indentation response as a probe indents the specimen [14]. AFM-based force spectroscopy was performed using an MFP-3D Infinity system (Asylum Research, Oxford Instruments) equipped with a fluid cell-lite chamber which is filled with PBS to maintain full hydration of the samples throughout measurement at room temperature. Figure 1 illustrates the complete experimental workflow and calibration process used in this study.

AFM measurements in this study required a fully hydrated, stable liquid environment to preserve physiological tissue properties throughout the experiment [7]. Figure 1a shows a schematic diagram of the AFM setup proper for liquid-environment measurements. The sample is immersed in PBS to maintain full hydration and physiological conditions during indentation. A rubber sealing ring (black) that covers the fluid cell minimizes evaporation throughout the experiment. The system uses a laser beam reflected from the back of the microcantilever, detected by a quadrant photodetector to measure deflections with nanometer precision.

Accurate mechanical characterization required a rigorous two-step calibration process. Figure 1b outlines the calibration workflow designed to ensure reliable stiffness measurements. First, the inverse optical lever sensitivity (InvOLS) was determined by acquiring force curves on a flat sapphire substrate, which served as an infinitely hard reference, assuming negligible indentation. The resulting approach curve displayed a steep, linear slope, allowing precise determination of InvOLS to convert the photodetector signal into cantilever deflection. The cantilever spring constant was then calculated using the thermal noise method [46], which accounts for probe-specific variability and is used to deduce the applied force from the measured deflection. After these steps, the tip radius was calibrated using a representative soft calibration sample—2.5% agarose gel with a known Young’s modulus [19, 36, 46].

Figure 1c presents representative deflection–displacement curves obtained on sapphire and agarose substrates in PBS. The sapphire approach curve exhibits a steep, nearly linear response upon contact, reflecting its rigid, non-deformable nature. In contrast, the agarose sample exhibits a more gradual, nonlinear response during approach and retraction, indicative of its compliant nature. Using the sapphire response as a zero-indentation reference, the deflection–displacement data for agarose were converted into a force–indentation curve, as shown in Figure 1d. To calibrate the cantilever’s tip radius, a 10 × 10 grid of force map was acquired over a 2 µm × 2 µm area on the 2.5% agarose gel with a known Young’s modulus of 254 kPa [36] as shown in Figure 1e. From the resulting 100 force–indentation curves, modulus values were extracted from the retraction segments using the Johnson–Kendall–Roberts (JKR) contact mechanics model [30], which accounts for adhesion and is well suited for soft biological materials [29]. The tip radius was iteratively adjusted to have the mean modulus across the grid matched the reference value of 254 kPa. Figure 1f displays a histogram of the extracted Young’s modulus values, showing a Gaussian-like peak centered at ~ 254 kPa with a standard deviation of 6.75 kPa [19]. The final calibrated tip radius obtained through this procedure was then applied to all subsequent mechanical measurements on lens capsule samples to ensure accurate and consistent modulus quantification. Across cantilevers, the calibrated tip radii ranged from approximately 80 nm to 2 µm. Such values are typical for effective contact radii obtained in AFM force spectroscopy of soft hydrated materials, where tip blunting, surface adsorbates, and liquid meniscus effects increase the fitted contact area beyond the nominal apex radius [30, 47–50]. Because anterior and posterior measurements for each pig were performed with the same calibrated cantilever under identical conditions, all regional comparisons rely on internally consistent relative measurements that are robust to variation in the absolute radius. This calibration approach builds on our prior AFM work characterizing nanomechanical properties across a wide range of biological systems, including polymer–cell interactions, bone tissue aging, collagen piezoelectricity, and viral structural damage, where similarly rigorous calibration protocols were employed [37–40].

**Fig. 2:**
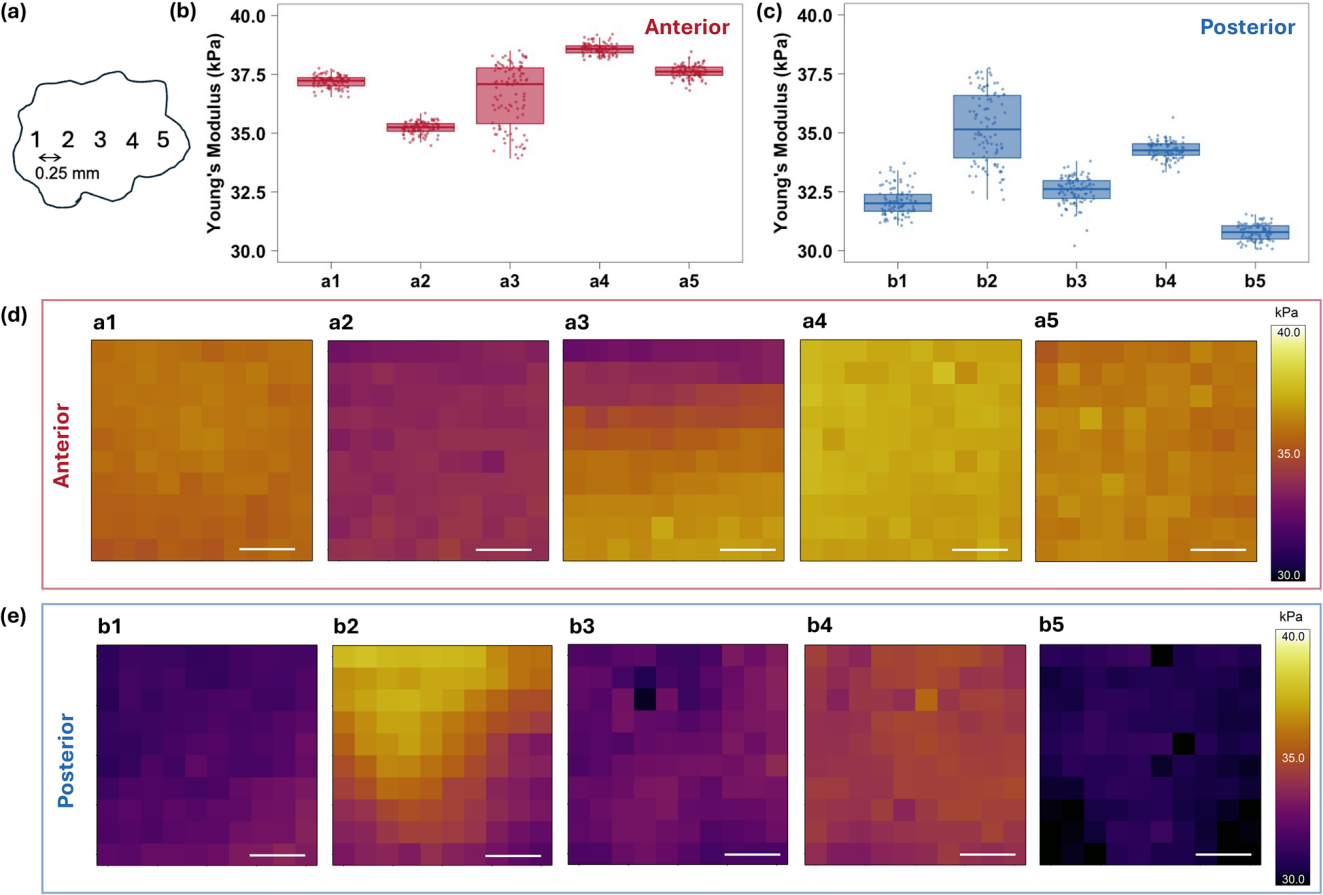
Experimental design and spatial mapping of Young’s modulus across anterior and posterior regions of the porcine lens capsule. **a** Schematic illustrating the measurement design with five discrete sites spaced 0.25 mm apart along the anterior and posterior surfaces of the same isolated capsule segment. Each site was mapped using AFM-based force spectroscopy in a 10 × 10 grid covering 2 µm × 2 µm. **b**,** c** Box plots of Young’s modulus values measured at the five positions for anterior (b, red) and posterior (c, blue) regions, respectively. Each point represents an individual force–indentation measurement. **d**, **e** Representative color-coded Young’s modulus maps at the five measurement sites for anterior (d) and posterior (e) regions, showing local microscale heterogeneity without consistent intra-regional differences. Anterior maps consistently exhibit higher median stiffness than posterior maps within the same sample. Scale bars = 500 nm

For lens capsule measurements, identical silicon cantilevers (AC240TS-R3, nominal spring constant 2 N/m, OLYMPUS) were used consistently across all experiments to ensure comparability. For the anatomical mapping shown in Figure 2, each anterior or posterior capsule was sampled at five predefined regions, with each region measured using a 10 × 10 force map over a 2 µm × 2 µm area. The approach velocity was fixed at 2 µm/s to minimize viscoelastic artifacts, yielding 100 force–indentation curves per region. For the repeated-measurement datasets shown in Figure 4, each capsule was additionally measured at multiple independent sites to assess within-sample reproducibility. A minimum of two and on average five spatially distinct sites were measured per sample, each acquired as a 10 × 10 force map under identical calibration and environmental conditions, producing approximately 500 curves per sample. This repeated-site design allowed us to evaluate microscale consistency across the capsule surface and ensured that observed regional differences reflected true biological heterogeneity rather than sampling variability. Across 13 paired porcine lens capsule samples (anterior–posterior pairs), this generated over 5000 curves per anatomical region. To enable accurate comparison between anterior and posterior regions, all measurements for each anterior–posterior pair were performed on the same day using the same calibrated cantilever to ensure fair comparison with the minimized influence of calibration variability.

### Data Analysis

Force–indentation curves were processed using Igor Pro software (Asylum Research), with additional analysis and statistical visualization conducted in R and MATLAB. Young’s modulus values were extracted from the retraction segment of each force curve using the JKR contact mechanics model. The JKR model is particularly well suited for soft, hydrated biological samples due to its ability to incorporate adhesive interactions, which are often significant in tissues such as the lens capsule. Fitting the retraction segment specifically helps minimize potential errors introduced by viscous drag and substrate approach artifacts, providing more reliable contact mechanics in compliant materials model [30].

Distributions of modulus values were summarized using descriptive statistics including the mean, median, standard deviation (SD), standard error of the mean (SEM), and 95% confidence intervals (CI). Normality was evaluated using the Shapiro–Wilk test [51] for each experimental set (*n* < 5000) and the Anderson–Darling test [52] for the pooled anterior and posterior datasets (*n* > 5000), all of which confirmed non-normal distributions. Because of this non-Gaussian behavior, we additionally report the interpercentile range (90th–10th percentile), a robust and distribution-free measure of overall variability that captures the spread of stiffness values in each region.

To evaluate differences between anterior and posterior regions, the Wilcoxon rank-sum test (Mann–Whitney U test) [53] was used because the Young’s modulus values were not normally distributed. Each experimental set consisted of an anterior capsule from one eye and a posterior capsule from the opposite eye of the same pig, which provides biological pairing at the level of the animal. However, the individual AFM indentation curves from the two regions were not obtained at corresponding anatomical locations, and the number of curves differed between groups. Because the measurements could not be treated as paired observations, the unpaired Wilcoxon rank-sum test was applied to compare the modulus distributions. Statistical significance was defined as *p* < 0.05. *Clinical trial number: not applicable.*

## Results

**Fig. 3:**
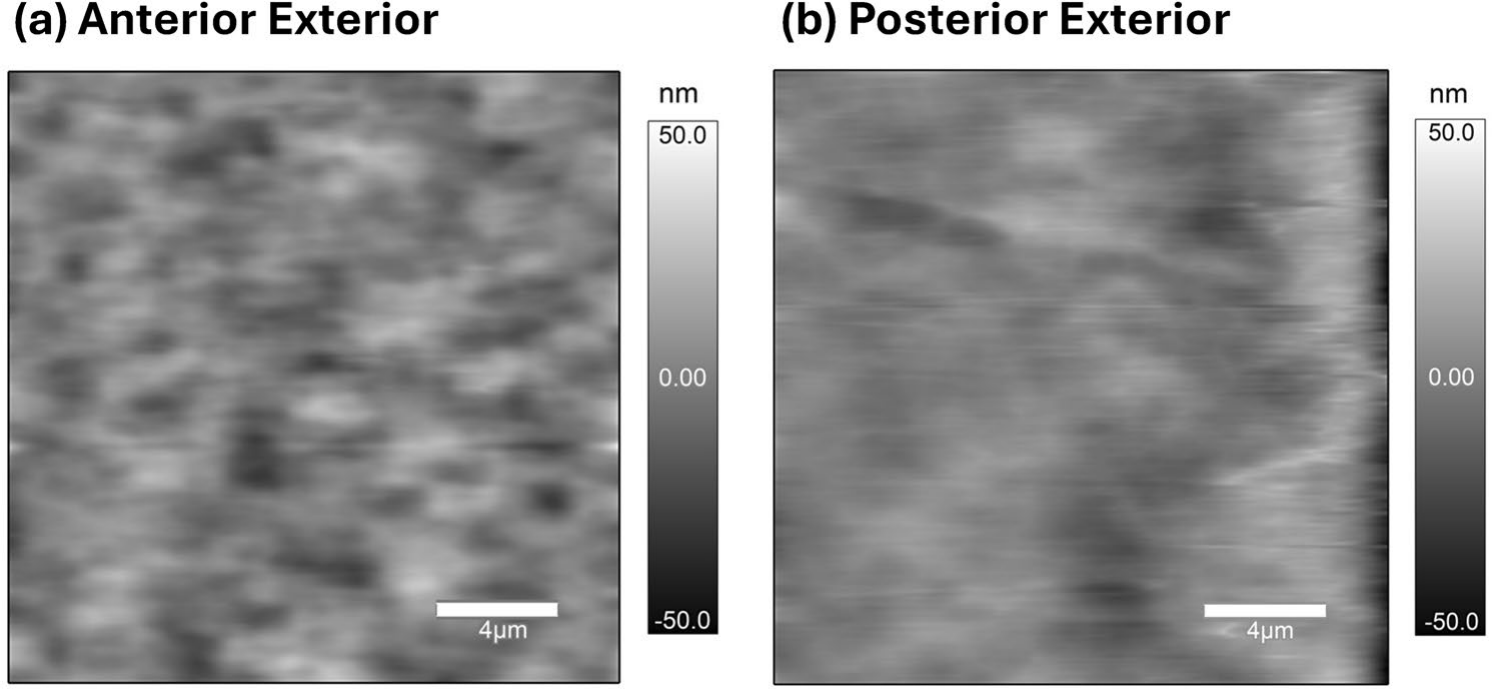
AFM topography of the anterior and posterior exterior regions of the porcine lens capsule. **a** Anterior and **b** posterior exterior surfaces exhibit relatively homogeneous morphologies without pronounced structural irregularities or artifacts. AFM height maps were acquired over 20 µm × 20 µm scan areas in liquid, with color scales indicating height variations from –50 nm to +50 nm. Both regions display generally uniform surface topographies that support the interpretation that observed stiffness differences arise from intrinsic matrix structure rather than surface roughness. Scale bars = 4 µm

To investigate regional mechanical variability within the porcine lens capsule, AFM-based force spectroscopy was performed at five discrete positions along both the anterior and posterior surfaces of each specimen. Measurement sites were spaced at 0.25 mm intervals along a linear axis (Figure 2a), and each position was mapped over a 2 µm × 2 µm area with a 10 × 10 grid, yielding 100 force–indentation curves per site to capture local stiffness distributions [29, 32]. Figure 2 shows the Young’s modulus values measured at these five positions for anterior (b,d) and posterior (c,e) samples in a representative porcine lens. Each 2 µm × 2 µm force map reveals microscale stiffness variations of approximately ± 5–10% relative to the mean modulus, highlighting subtle within-sample variability. Anterior sites (a1–a5) exhibited median modulus values ranging from 33.8 to 37.1 kPa, with site-specific standard deviations between 0.23 and 1.22 kPa. Across all anterior positions, values spanned from 32.7 to 37.7 kPa, yielding an overall mean of 35.6 kPa. Posterior sites (b1–b5) showed consistently lower median values of 30.7 to 35.1 kPa, with standard deviations of 0.36 to 1.49 kPa, spanning an overall range of 29.1 to 37.7 kPa with a mean of 33.0 kPa. No systematic trend along was observed along the measurement axis, indicating no consistent intra-regional difference at this spatial scale. However, anterior maps consistently exhibited higher stiffness compared to posterior maps. These spatially resolved measurements demonstrate that, although both regions show local microscale variation without a clear positional trend, the anterior capsule is consistently stiffer than the posterior within the same biological sample. This intrinsic regional distinction suggests underlying differences in matrix organization and composition, reinforcing the value of AFM-based force mapping for detecting subtle but consistent biomechanical heterogeneity in soft biological tissues. Although two sites, A3 and B2 in Fig. 2d and e, exhibited greater local heterogeneity than the other locations, the AFM topographic images did not show corresponding surface irregularities (Fig. 3). Prior studies have shown that the lens capsule contains natural microscale variation in collagen IV network density and basement membrane organization [1, 44], and the increased variability at these sites is therefore most likely attributable to intrinsic matrix heterogeneity rather than an imaging artifact. This localized variability occurred at only one anterior and one posterior site and did not affect the overall regional comparison.

To evaluate whether the observed stiffness differences between anterior and posterior regions could be influenced by surface morphology, AFM topographic scans were acquired over larger 20 µm × 20 µm areas from both regions of the same specimen. These scans were performed in PBS using the same silicon cantilevers (AC240TS-R3, Olympus; nominal spring constant ~ 2 N/m) operated in tapping mode. Fig. 3a and b displays representative height maps for the anterior and posterior surfaces, respectively, with color scales indicating height variations of approximately ± 50 nm. Both regions exhibited relatively smooth and homogeneous topographies without pronounced structural irregularities or mounting artifacts that could confound indentation measurements. This is consistent with prior AFM studies on biological membranes and cells, which emphasize the importance of controlling for surface roughness in mechanical analysis.^17^ Recent dynamic AFM topography–mechanics correlation studies further highlight how nanoscale surface morphology can influence stiffness interpretation in live biological samples [17]. The observed uniform morphology across anterior and posterior regions supports the interpretation that the measured regional differences in Young’s modulus primarily reflect intrinsic differences in matrix structure and composition, rather than variations in surface roughness. This analysis strengthens the conclusion that the stiffness difference identified by AFM-based force mapping is a true material property difference between anterior and posterior capsule regions.

**Fig. 4:**
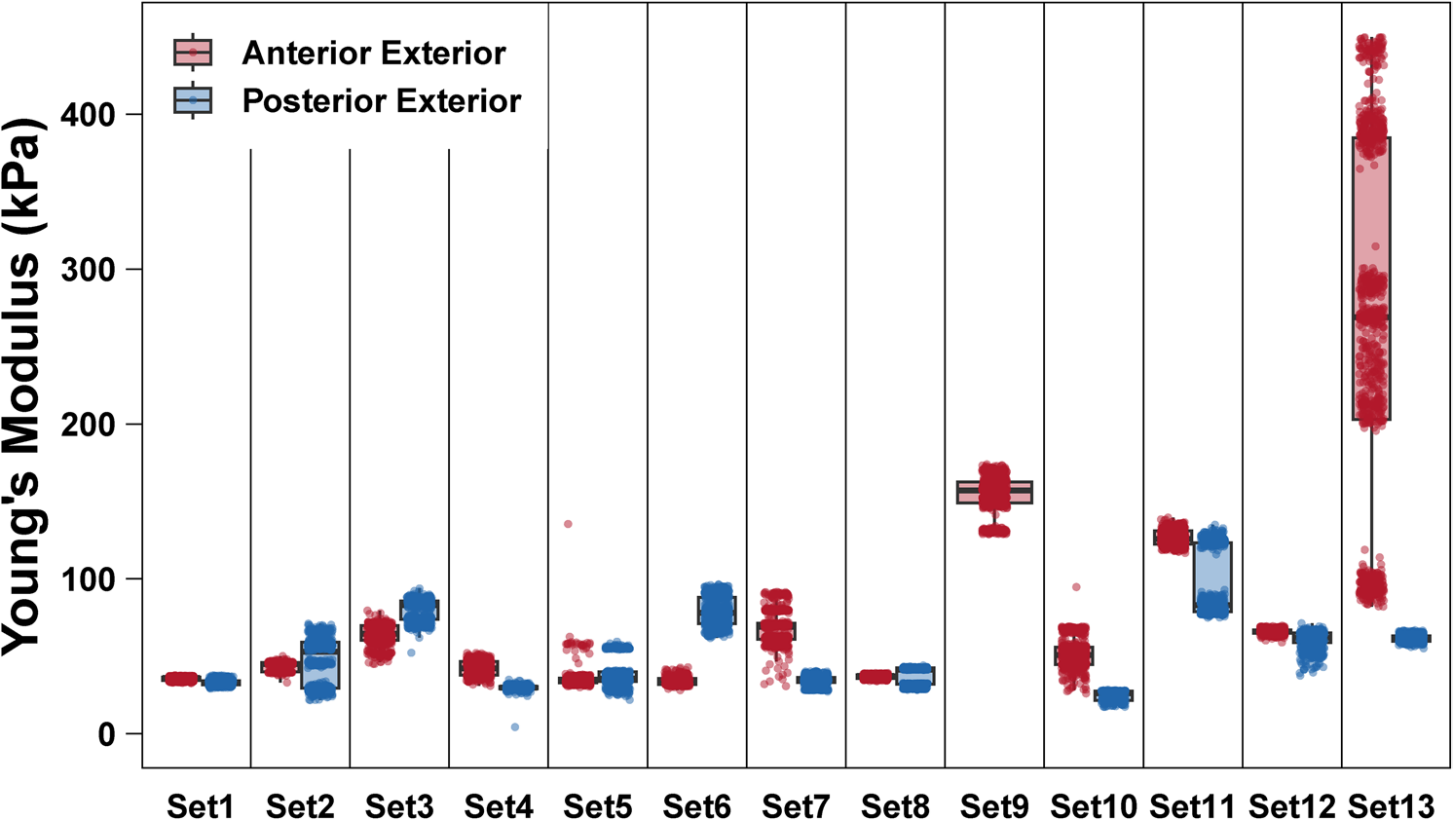
Comparison of Young’s modulus across 13 experimental sets for anterior and posterior regions of the porcine lens capsule. Box plots show anterior (red) and posterior (blue) Young’s modulus values measured across 13 independent AFM experiments using a consistent calibration protocol. Most datasets exhibit higher anterior stiffness, supporting a robust anterior–posterior difference. Notably, Set 13 shows anomalously high stiffness and larger variation in the anterior sample compared to other sets, suggesting possible biological variability or measurement artefact

To assess the reproducibility and robustness of the observed anterior–posterior stiffness differences, AFM-based force spectroscopy was performed across 13 independent experimental sets, each conducted on separate days using the same calibration workflow and measurement parameters. Figure 4 presents box plots summarizing Young’s modulus values from anterior (red) and posterior (blue) exterior regions across these 13 datasets (Set 1–Set 13). Each set represents paired measurements from the same porcine lens capsule, acquired with identical cantilevers and calibration parameters to ensure experimental consistency.

Across the majority of datasets, the anterior region exhibited higher median Young’s modulus values than the posterior region. Anterior measurements also displayed greater variability, reflected in wider interquartile ranges and larger standard deviations. This pattern is consistent with the regional differences observed in spatial force mapping (Fig. 2) and supports the conclusion that the anterior capsule possesses intrinsically higher stiffness at micrometer scales.

While most sets followed this trend, a few deviations were observed. In Sets 2, 3, and 6, the posterior region exhibited slightly higher stiffness than the anterior, indicating possible effects of local tissue variation, sample orientation, or subtle differences in preparation. Set 13, in contrast, showed an unusually high modulus in the anterior region relative to all other sets, while the posterior values remained within the expected range. This abnormal elevation may reflect a measurement artifact, mounting issue, or localized biological anomaly. Calibration checks and the quality of the force–indentation curves in Set 13 were consistent with all other datasets, suggesting that this elevated variability reflected genuine biological heterogeneity rather than a measurement artifact.

This dataset-level reproducibility highlights the importance of the rigorous two-step calibration protocol employed in this study, involving sapphire and agarose reference materials and JKR model fitting applied to the retraction segment of force–indentation curves. Such calibration minimizes inter-experimental variability and ensures comparability across sessions. Comparable calibration and contact-model approaches have been applied successfully in hydrated tissue stiffness mapping using AFM, ensuring accuracy and comparability across datasets [21]. The use of the JKR model, which incorporates adhesive effects relevant in hydrated biological samples, further enhances the accuracy and physiological relevance of modulus estimation. Together, these results confirm that the anterior–posterior stiffness difference is a reproducible and robust biomechanical feature of the porcine lens capsule.

**Fig. 5:**
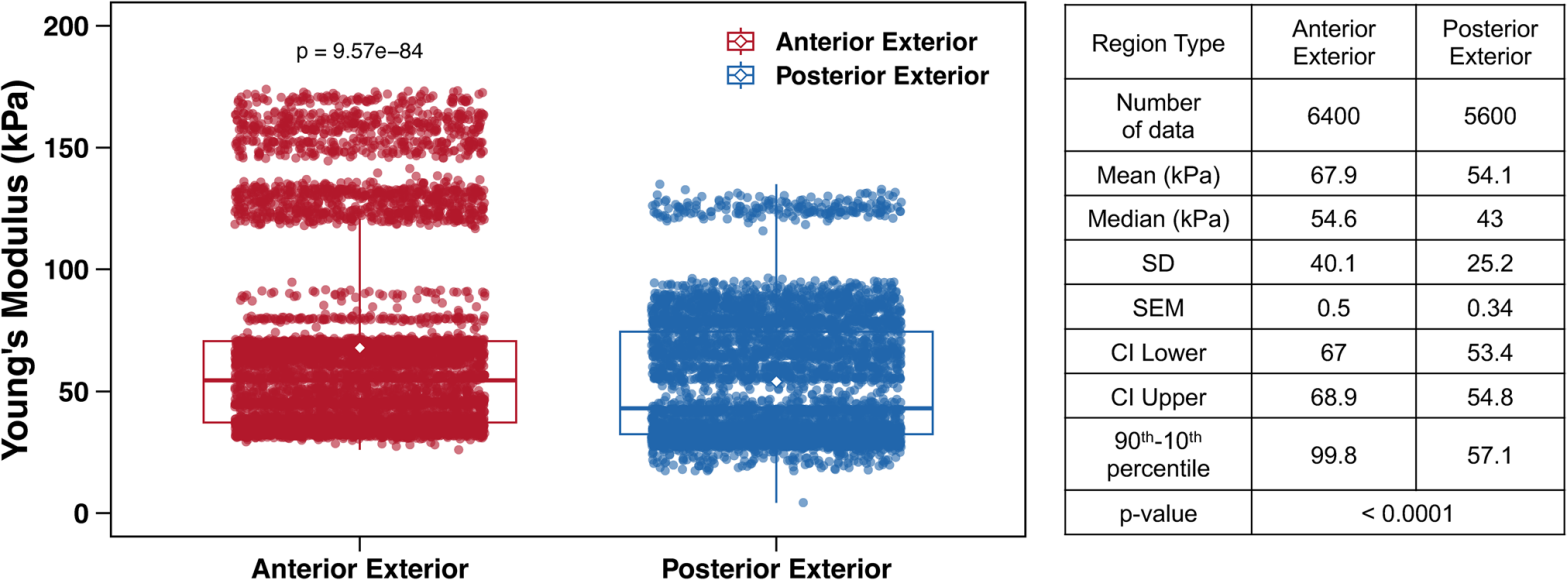
Comparison of Young’s modulus between anterior and posterior exterior regions of the porcine lens capsule. Box plots summarize force–indentation data pooled from 12 experimental datasets (excluding Set 13 due to anomalous anterior values), with anterior (red, *n* = 6400) and posterior (blue, *n* = 5600) measurements. The anterior region exhibits significantly higher modulus (mean 67.9 kPa, median 54.6 kPa) than the posterior (mean 54.1 kPa, median 43 kPa), with greater variability. The difference was statistically significant (Wilcoxon rank-sum test, *p* < 0.0001), confirming a robust regional stiffness difference

To evaluate overall regional differences in mechanical properties, all force–indentation measurements from anterior and posterior regions were pooled for statistical analysis. Figure 5 presents box plots comparing Young’s modulus values from the anterior (*n *= 6400) and posterior (*n* = 5600) regions of the porcine lens capsule, excluding Set 13 due to its anomalously elevated anterior values. Set 13 was removed from this pooled comparison to prevent its unusually large variability from disproportionately influencing the combined distribution. The Wilcoxon rank-sum test [53] confirmed that this difference was highly statistically significant (*p* < 0.0001), indicating that the distribution of the two groups is distinct. Specifically, anterior regions exhibited significantly higher stiffness, with a mean modulus of 67.9 kPa and a median of 54.6 kPa, compared to a mean modulus of 54.1 kPa and a median of 43 kPa for posterior regions. Additionally, anterior measurements displayed greater variability, with a standard deviation of 40.1 kPa compared to 25.2 kPa in the posterior region. This difference was further reflected in the 90th-10th percentile range, which was markedly wider in the anterior capsule (99.8 kPa) than in the posterior capsule (57.1 kPa), consistent with the extended upper tail visible in the jitter distribution (Fig. 5). These findings are consistent with prior bulk mechanical testing studies that reported an anterior–posterior stiffness difference [5, 19, 24], underscoring the physiological relevance of regional biomechanical specialization in the lens capsule. The AFM-based approach used here further improves spatial resolution under hydrated, physiologically relevant conditions. AFM-based modulus mapping has been similarly applied to extracellular matrix and cell–substrate systems, revealing that nanoscale structural organization and surface topography can strongly modulate local stiffness [22]. By capturing thousands of measurements across multiple sites and controlling for outlier datasets, this study provides a robust, detailed characterization of regional mechanical differences that likely reflect variations in collagen architecture, matrix cross-linking, and developmental biology of the lens capsule.

## Discussion

The mechanical differences observed between the anterior and posterior regions of the porcine lens capsule likely reflect fundamental distinctions in their developmental origins, matrix composition, and functional demands. The anterior capsule thickens progressively throughout life due to continuous extracellular matrix secretion by lens epithelial cells, resulting in a collagen-rich, densely organized structure [1, 2]. This composition imparts mechanical robustness to the anterior capsule, enabling it to withstand both physiological forces generated during accommodation and stresses encountered during surgical procedures. In contrast, the posterior capsule lacks an epithelial layer and ceases substantial matrix synthesis after birth, maintaining a thinner and more compliant profile [3, 18]. The anterior capsule is structurally thicker, collagen-denser, and remains biologically active throughout life due to its epithelial covering [1, 3, 13], whereas the posterior capsule lacks an epithelial layer and ceases substantial matrix production after early development [3, 18], providing a structural basis for the consistently higher stiffness observed in the anterior region. These intrinsic differences in matrix structure are consistent with the higher stiffness and micrometer-scale heterogeneity observed in the anterior region through AFM-based force spectroscopy. Given that these measurements were performed on young porcine lenses, it is reasonable to speculate that such anterior–posterior differences would be even more pronounced in aging human lens capsules, where progressive stiffening and thickening of the anterior capsule contribute to presbyopia and increased surgical risk.

Furthermore, the anterior capsule is chronically exposed to larger variations in environmental stimuli such as light, oxidative stress, and dynamic aqueous-humor flow. These factors may induce spatially heterogeneous remodeling over time, including localized collagen cross-linking or microstructural stiffening. Functionally, the anterior capsule bears greater biomechanical load during accommodation and experiences continuous exposure to aqueous-humor metabolites such as glucose, conditions that may promote collagen cross-linking and contribute to its increased stiffness relative to the posterior capsule [4, 8, 10]. Such environment- and matrix-driven remodeling is consistent with broader evidence showing that extracellular matrix composition and mechanics actively influence tissue function and adaptation [35]. This hypothesis is supported by the consistently observed mechanical variability across anterior maps, suggesting a complex interplay between developmental programming and cumulative environmental stress.

These biomechanical properties may enhance the anterior capsule’s resilience during procedures such as capsulorhexis in cataract surgery, where capsular integrity is critical for surgical success [6]. In contrast, the posterior capsule’s relative compliance may contribute to its vulnerability to deformation, rupture, or postoperative complications such as posterior capsule opacification [5]. These regional mechanical differences have direct translational relevance, as accurate knowledge of anterior–posterior stiffness contrast can improve finite-element models of capsular-bag dynamics [11], inform the design of accommodative intraocular lenses, and guide surgical strategies that minimize stress concentrations and reduce the risk of posterior capsular rupture [5, 14–16]. Importantly, the challenges of resolving such regional heterogeneity are mirrored in other biological systems. For example, AFM and continuum approaches have revealed spatially varying mechanical properties in breast tissue [27], cartilage [28], and ocular basement membranes such as Bowman’s membrane [34].

The high-resolution AFM-based approach employed in this study enabled precise quantification of micromechanical heterogeneity under fully hydrated, physiologically relevant conditions. By capturing thousands of localized measurements, this methodology revealed subtle regional variations that conventional bulk mechanical tests might overlook [19, 24], offering deeper insights into lens capsule biomechanics.

A deeper understanding of micrometer-scale mechanical properties can inform the design of accommodative intraocular lenses (IOLs) and surgical techniques that minimize mechanical mismatch with the native capsule, thereby reducing postoperative complications and enhancing IOL performance. Collectively, the AFM-based force spectroscopy framework established here provides a powerful analytical tool for characterizing micromechanical behavior in ocular tissues and may facilitate future studies on aging, disease progression, and the mechanical impacts of surgical interventions. Moreover, integrating AFM-based mapping with biochemical analyses and ultrastructural imaging could offer a more comprehensive understanding of the structure–function relationships in the lens capsule, particularly in the context of age-related and pathological changes.

This study provides a detailed mechanical characterization of the porcine lens capsule, focusing on regional differences between the anterior and posterior exterior surfaces using AFM in a hydrated, physiologically relevant environment. High-resolution force mapping combined with rigorously calibrated indentation analysis revealed that the anterior capsule exhibits significantly greater stiffness and mechanical heterogeneity compared to the posterior region. These differences were robust across multiple datasets and measurement days, supported by rigorous calibration protocols and consistent AFM operation. Furthermore, these differences likely reflect variations in structural composition, developmental processes, and functional demands inherent to each region.

Topographic analyses further confirmed that surface morphology did not confound the mechanical measurements, reinforcing that the observed modulus difference is an intrinsic material property. These findings align with the known biological and developmental differences between the two regions: the anterior capsule is thicker, structurally denser, and more biologically active, whereas the posterior capsule is thinner and relatively quiescent post-development.

By capturing thousands of localized force–indentation curves, this approach provided a detailed mechanical map of the lens capsule, offering insights not accessible through bulk mechanical testing. These results underscore the utility of AFM-based characterization for assessing tissue biomechanics at nanoscale resolution and have potential translational implications for improving intraocular lens design and surgical strategies in cataract treatment.

Future research integrating AFM-based mechanical mapping with histological or ultrastructural imaging of anterior and posterior regions will be essential for correlating microscale mechanical differences with underlying matrix architecture and for advancing understanding of pathological remodeling such as posterior capsular opacification [44]. Future research integrating mechanical, biochemical, and ultrastructural data will further elucidate the structure–function relationships in the lens capsule, particularly in the context of aging, disease, and surgical intervention.

## Supplementary Information

Below is the link to the electronic supplementary material.

Supplementary file1 (DOCX 265 KB)

## Data Availability

The datasets generated and analyzed during the current study are available from the corresponding author upon reasonable request.
