# Supplementary material for: Regional Differences in Young’s Modulus of the Porcine Lens Capsule
Source: Ann Biomed Eng. 2026 Feb 14;54(7):2027–39. doi: 10.1007/s10439-026-04012-0 (PMC13131290; doi:10.1007/s10439-026-04012-0)
Supplement: Supplementary file 1 — Supplementary file1 (DOCX 265 KB) [file 10439_2026_4012_MOESM1_ESM.docx]

**Supplementary Information**

**Regional Differences in Young’s Modulus of the Porcine Lens Capsule**

Annals of Biomedical Engineering

Hoyeon Jang^1^, Myles Cline^1^, Jeongjin Lee^2^, Matthew Reilly^1^, Hanna Cho^3^*

^1^ Department of Biomedical Engineering, ^2^ Division of Biostatistics, ^3^ Department of Mechanical and Aerospace Engineering

The Ohio State University, Columbus, Ohio 43210, United States

Email: cho.867@osu.edu

S1. Normality test for each experimental set

Normality was assessed separately for the anterior and posterior regions within each experimental set using the Shapiro–Wilk test, which is appropriate for sample sizes up to 5000. A full summary of p-values and sample sizes for all sets is provided in Table S1. Across sets, most modulus distributions showed statistically significant deviations from normality.

Table S1. Shapiro–Wilk normality test results for each experimental set.

| **Set** | **Region Type** | **n** | **Shapiro_p** |
| --- | --- | --- | --- |
| Set 1 | Anterior Exterior | 500 | 5.86E-16 |
|  | Posterior Exterior | 500 | 8.05E-08 |
| Set 2 | Anterior Exterior | 300 | 7.77E-17 |
|  | Posterior Exterior | 600 | 8.66E-21 |
| Set3 | Anterior Exterior | 500 | 2.38E-14 |
|  | Posterior Exterior | 500 | 3.04E-17 |
| Set4 | Anterior Exterior | 500 | 1.19E-10 |
|  | Posterior Exterior | 400 | 3.44E-27 |
| Set5 | Anterior Exterior | 600 | 6.81E-40 |
|  | Posterior Exterior | 500 | 1.15E-21 |
| Set6 | Anterior Exterior | 300 | 1.62E-12 |
|  | Posterior Exterior | 700 | 5.25E-14 |
| Set7 | Anterior Exterior | 700 | 3.80E-15 |
|  | Posterior Exterior | 600 | 1.70E-16 |
| Set8 | Anterior Exterior | 500 | 1.66E-15 |
|  | Posterior Exterior | 500 | 5.44E-27 |
| Set9 | Anterior Exterior | 700 | 1.41E-19 |
| Set10 | Anterior Exterior | 700 | 1.99E-14 |
|  | Posterior Exterior | 200 | 1.63E-13 |
| Set11 | Anterior Exterior | 600 | 5.57E-12 |
|  | Posterior Exterior | 600 | 2.01E-30 |
| Set12 | Anterior Exterior | 500 | 1.66E-3 |
|  | Posterior Exterior | 500 | 2.49E-19 |
| Set13 | Anterior Exterior | 900 | 6.68E-22 |
|  | Posterior Exterior | 500 | 2.67E-06 |

Figure S1. Density scaled histograms for each experimental set (anterior and posterior). To visualize the distributional patterns revealed by the normality tests, density histograms were generated for each experimental set (Sets 1-13). These plots show the distribution of Young’s modulus values for the same dataset used in Figure 4 of the main text, with anterior and posterior regions displayed together for direct comparison. Set 9 includes only anterior data because no posterior measurement was available.

S2. Normality assessment for pooled anterior and posterior datasets

To complement the set specific normality assessments presented in S1, we evaluated normality at the global level by pooling all anterior modulus values across Sets 1 through 13 into a single dataset and performing the same analysis for the posterior values. Because the pooled sample sizes exceeded the valid range for the Shapiro–Wilk test, we used the Anderson–Darling test, which is suitable for large samples. Both pooled distributions deviated significantly from normality (anterior: A^2^ = 761.92, p < 0.001; posterior: A^2^ = 166.17, p < 0.001), indicating that the stiffness values remain strongly non normal even when aggregated across all sets.

To visualize the pooled distributional characteristics, histograms for the anterior and posterior modulus values are shown in Figure S2. Both distributions exhibit right skewed and heavy tailed shapes, consistent with the micrometer scale heterogeneity observed in the individual AFM force maps.

Figure S2. Pooled histograms for anterior and posterior Young’s modulus values.

**S3. Wilcoxon rank sum comparison across experimental sets**

**Although the anterior and posterior capsules from each pig constitute a biological pair in the experimental design, the AFM indentation curves from the two regions were not anatomically matched. Therefore, a paired test was not appropriate, and the unpaired Wilcoxon rank sum test was used. The test was applied separately to each experimental set, and all twelve evaluable sets showed extremely small p-values (all p < 10^-8^), demonstrating that the anterior capsule is significantly different from the posterior capsule in every sample. Table S3 summarizes the results.**

**Table S3. Wilcoxon rank sum test comparing anterior and posterior Young’s modulus distributions for each experimental set. Set 9 is omitted because only anterior data were available.**

| **Set** | **n_Anterior** | **n_Posterior** | **Wilcoxon_p** |
| --- | --- | --- | --- |
| Set 1 | 500 | 500 | 7.06E-90 |
| Set 2 | 300 | 600 | 7.96E-8 |
| Set 3 | 500 | 500 | 1.02E-130 |
| Set 4 | 500 | 400 | 2.19E-146 |
| Set 5 | 600 | 500 | 8.77E-11 |
| Set 6 | 300 | 700 | 6.87E-139 |
| Set 7 | 700 | 600 | 4.11E-209 |
| Set 8 | 500 | 500 | 4.39E-8 |
| Set 10 | 700 | 200 | 6.61E-103 |
| Set 11 | 600 | 600 | 5.06E-99 |
| Set 12 | 500 | 500 | 3.24E-74 |
| Set 13 | 900 | 500 | 1.47E-211 |
